# Effect of Induced Membrane Formation Followed by Polymethylmethacrylate Implantation on Diabetic Foot Ulcer Healing When Revascularization Is Not Feasible

**DOI:** 10.1155/2019/2429136

**Published:** 2019-11-19

**Authors:** Chao Liu, Jia-Xing You, Yi-Xin Chen, Wei-Fen Zhu, Ying Wang, Pan-Pan Lv, Feng Zhao, Hong-Ye Li, Lin Li

**Affiliations:** ^1^Department of Orthopedics, Zhejiang University School of Medicine Sir Run Run Shaw Hospital, #3 E. Qingchun Rd, Hangzhou 310016, China; ^2^Department of Endocrinology, Zhejiang University School of Medicine Sir Run Run Shaw Hospital, #3 E. Qingchun Rd, Hangzhou 310016, China; ^3^Wound and Ostomy Care Clinic, Zhejiang University School of Medicine Sir Run Run Shaw Hospital, #3 E. Qingchun Rd, Hangzhou 310016, China; ^4^Department of Ultrasound, Zhejiang University School of Medicine Sir Run Run Shaw Hospital, #3 E. Qingchun Rd, Hangzhou 310016, China; ^5^Department of Clinical Laboratory, Zhejiang University School of Medicine Sir Run Run Shaw Hospital, #3 E. Qingchun Rd, Hangzhou 310016, China

## Abstract

No study has investigated the role of induced membrane (IM) formation in treating diabetic foot ulcer (DFU). This retrospective study was aimed (1) at evaluating the potential role of a two-staged surgical approach, comprising polymethylmethacrylate (PMMA) implantation and IM formation, in the treatment of DFU and (2) at comparing the results of those with routine wound debridement in patients with DFUs and nonrevascularized peripheral arterial disease (PAD). Fifty patients with infected DFUs who were not candidates for vascular interventions were enrolled between February 2016 and April 2018 and assigned to the PMMA group (*n* = 28) and conventional group (*n* = 22). The healing rate, major amputation rate, duration of healing, frequency of debridement procedures, patient survival rate, and reulceration of DFUs were determined. The Mann-Whitney *U* test, independent sample *t*-test, and *χ*^2^ or Fisher exact test were used in statistical analysis. Overall clinical outcomes were statistically different between the groups (*Z* = −2.495, *P* = 0.013). In the PMMA group, 16 patients (57.1%) with intact IM formation achieved ulceration healing at 13.1 ± 3.7 weeks with a mean number of debridements of 1.3 ± 0.4, which were significantly different compared to those values in 5 patients of the conventional group (22.7%, *P* = 0.014; healing duration: 26.4 ± 7.8 weeks, *P* = 0.016; mean number of debridements: 3.6 ± 0.5, *P* ≤ 0.001). At a mean 16.8 ± 4.3-month follow-up, patient survival rates were 92.9% and 68.2% in the PMMA and conventional groups, respectively (*P* = 0.032). The major amputation rate and reulceration of DFUs were similar between the groups. The two-staged surgical approach is an available, effective modality for improving healing of DFUs. This study provides preliminary information of IM formation followed by PMMA implantation in the management of DFUs in PAD when revascularization is not feasible.

## 1. Introduction

Diabetic foot ulcer (DFU) is one of the most feared complications with a lifetime incidence of 15-25% in patients with diabetes mellitus [1, 2]. More than half of DFUs are clinically infected on initial presentation at a clinic, which precedes 85% of all cases of nontraumatic amputation of the lower limbs [2, 3]. Peripheral arterial disease (PAD) is an important precipitating factor for the development of ulceration and limb loss. Arterial insufficiency in the foot resulting from PAD not only substantially compromises the healing process of DFUs but also independently increases the risk of infection, amputation, and mortality [4, 5]. It is currently estimated that at least 50% of patients with DFUs have an ischemic component, among whom the 5-year survival has been shown to be as low as 50% and the 2-year mortality is 50% following a major amputation [6–9].

Since PAD is associated with a notably poor prognosis, guidelines from diabetic and vascular societies recommend revascularization by either surgical bypass or endovascular therapy when a major amputation is under consideration in patients with an infected and hard-to-heal DFU because of PAD [10–13]. The 1-year limb salvage rate after vascular intervention ranges from 78% to 85%, which is markedly improved compared to that in patients without revascularization [7]. Although dedicated management of limb ischemia has indeed resulted in a decreased number of major amputations, it should be noted that up to one-third of patients with PAD are not suitable for revascularization surgery because of unfavorable vascular involvement, technical difficulty, or patient refusal. Currently, the treatment option is limited, and no Food and Drug Administration-approved products are indicated for treating patients with DFU and nonrevascularized PAD. Cellular therapy appears promising in this field. Meta-analyses revealed encouraging results in terms of accelerating wound healing, improving the healing rate, and reducing the amputation rate in these “no-option” patients [14, 15]. However, clinical trials on cellular therapy are still ongoing, and more experience with this treatment is eagerly awaited [16]. Furthermore, the high technical demand on cellular preparation and expensive medical cost also preclude its application in developing countries, such as China and India. Therefore, major amputation is often considered the ultimate solution if an infected DFU causes significant morbidity or threatens the survival of the patient [10, 17].

Local antibiotics have been proven effective for the treatment and prophylaxis of infection in orthopedic interventions [18–20]. With a key advantage of high drug concentration on the target location and low systemic toxic risks, topical antibiotic therapy serves as an available modality, especially in the avascular area. Polymethylmethacrylate (PMMA) cement is the major representative vehicle for local antibiotic delivery. Masquelet et al. [21] first discovered that the additional product from PMMA implantation, named the induced membrane (IM), is of great value for the reconstruction of segmental diaphyseal defects with nonvascularized bone autograft in a two-staged surgical protocol. Further histopathological analysis has revealed that this unique membrane is biologically active with inherent angiogenesis and potential osteogenic properties [22–25]. Although topical antibiotic therapy has gained popularity for treating DFU, we are not aware of any study concerning the role of IM in this subject. Therefore, the purposes of this study were (1) to assess the potential role of IM formation followed by antibiotic-loaded PMMA implantation in the treatment of DFUs and (2) to compare the results between this two-staged surgical protocol and conventional debridement in patients with hard-to-heal DFUs because of PAD. We hypothesized that the IM formation would be beneficial for wound healing and avoidance of major amputation when revascularization is not feasible.

## 2. Patients and Methods

### 2.1. Study Design

This study was conducted in a tertiary hospital, approved by the Human Research Ethics Committee of our affiliated university hospital, and adhered to the principles of the Declaration of Helsinki for medical research involving humans. Between February 2016 and April 2018, the medical records of patients with a deep DFU accompanied by an abscess, osteomyelitis, or forefoot gangrene (Wagner grade 3 or grade 4 severity) for a duration of more than 3 months, in combination with PAD (ankle-brachial index (ABI) < 0.9), who were not suitable for revascularization surgery were retrospectively evaluated. Patients were excluded if they were severely frail with unacceptable surgical risks or a short life expectancy (<3 months).

All the participants gave their written informed consent before the treatment and were stratified into two groups according to the type of surgical strategy. The conventional group included patients who were treated with regular wound debridement, and the PMMA group included those receiving a two-staged approach comprising PMMA implantation and IM formation. Besides different surgical interventions, the medical care in both groups was the same preoperatively and postoperatively, and the protocols based on the International Consensus on the Diabetic Foot [26], including blood glucose regulation, perfusion improvement by prostaglandins or antiplatelet drugs, postoperative dressings, and offloading, were followed. The antibiotic therapy was first managed empirically and then modified according to the results of an antibiogram from the intraoperative culture. All patients were regularly tracked by our multidisciplinary team, until death or the end of follow-up (May 2019).

### 2.2. Data Collection

Patient information, including sex, age, body mass index, type and duration of diabetes mellitus, duration and severity of DFU, ABI, active use of tobacco, and alcohol abuse, was abstracted through a clinical chart review by one of two reviewers not involved in the patient's care. Other relevant data, including laboratory findings and comorbidities, were also collected at initial admission.

### 2.3. Surgical Strategy

#### 2.3.1. Routine Debridement for the Conventional Group

General, spinal, or regional anesthesia was given to the patients depending on the anesthesiologist, and a tourniquet was not required. During the operation, nonviable and infected soft tissues were excised and debrided. The removal of infected bone was planned based on the preoperative radiograph, but the final decision was made according to the intraoperative findings. The edges of debridement were reached until the soft tissue and bone appeared macroscopically normal. After primary debridement, the skin was closed loosely, and the gauze dressings were changed once a day while the patient was in the ward. If the wound condition did not allow for primary closure, the negative pressure wound therapy system (VSD Medical Science and Technology Co. Ltd., Wuhan, China) was applied. With medical-grade polyurethane foam, this system sealed the wound with adhesive drapes and created a closed microenvironment by the connector tube connected to a wall-mounted suction device.

Patients underwent serial debridement at weekly intervals until there were no clinical signs and symptoms of infection. At that time, the wound was closed with 3-0 or 2-0 nylon stitches.

#### 2.3.2. Surgical Protocol for the PMMA Group

During the first stage, the initial debridement was performed in the same manner as for the conventional group. Subsequently, the defect created by debridement was filled with PMMA premixed with gentamycin (Cemex^Ⓡ^ Genta, Tecres Spa, Verona, Italy). A dose of 2 g vancomycin powder (Eli Lilly Japan K.K., Seishin Laboratories, Kobe, Japan) was added per package of PMMA for local depot delivery. When the cement was semirigid but still plastic, it was inserted into the defect to allow proper sizing and shaping of the spacer. During the last period of polymerization, the cement spacer was temporarily removed to avoid exothermic heating of the surrounding tissues. In an effort to eliminate any residual dead space, the PMMA spacer should be as large as possible; thus, primary wound closure was usually difficult to achieve. Patients were discharged from the hospital when medically and surgically stable, and the gauze dressings were changed at weekly intervals for two successive visits in the outpatient department.

**Figure 1:**
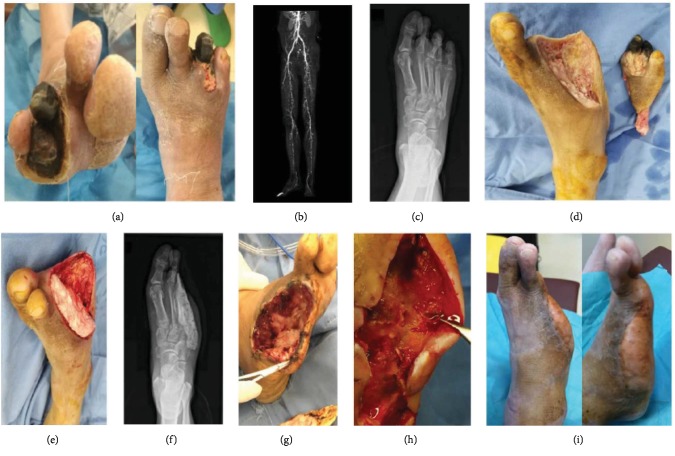
The two-staged surgical protocol for the polymethylmethacrylate (PMMA) group: (a) a 74-year-old male patient with diabetic foot ulceration (Wagner grade 4) in the right foot; (b) computed tomography angiography indicated peripheral arterial disease and revascularization surgery failed owing to calcific disease in the distal vasculature; (c) plain radiography showed osteomyelitis on the third, fourth, and fifth toes and corresponding metatarsal bones; (d) the nonviable, infected soft tissues and necrotic toes were debrided during the initial procedure; (e) the defect created by debridement was filled with vancomycin-loaded PMMA; (f) plain radiography showed the PMMA spacer at 3 days; (g) the block of the PMMA spacer was carefully exposed and removed after 3 weeks; (h) the intact induced membrane was a thin, semitransparent pseudosynovial membrane during the second procedure; (i) the ulcer was completely healed without signs and symptoms of osteomyelitis at follow-up.

During the third week after the initial procedure, patients returned to our day surgery unit for the second stage of intervention under local anesthesia. The previous incision at the defect site was reopened, and the block of the PMMA spacer was exposed and removed directly. The IM was determined by inspection and probing, without dissecting it from the surrounding tissues. If the IM was detectable and remained intact, the skin was closed together with the IM as one layer (Figure 1). Otherwise, further debridement was conducted in the same manner as for the first stage, and a new vancomycin-loaded PMMA was implanted.

### 2.4. Outcomes

The primary outcomes were the healing rate and major amputation rate. Healing was determined to be complete epithelialization of the surgical wound at two consecutive clinic visits. Nonhealing was defined as no significant reduction in the wound size or no significant decrease or worsening of secretions, with no need or refusal for major amputation. Major amputation (above the ankle) resulting from a life-threatening limb during the treatment period was considered to be failure of limb salvage.

The second outcomes included the duration of healing (the number of weeks from the initial surgical intervention to the date of complete healing), frequency of debridement procedures, patient survival rate, and reulceration (the appearance of a new ulcer at the same or contralateral foot during the follow-up).

### 2.5. Statistical Analysis

The results are presented as the mean ± standard deviation for quantitative variables and as absolute frequencies and percentages for categorical variables. Overall clinical outcomes were determined by the Mann-Whitney *U* test. In the comparison of separate primary and secondary outcomes, the independent sample *t*-test was used to analyze quantitative variables, and the *χ*^2^ test or Fisher exact test was used to analyze categorical variables. Statistical analyses were conducted with SPSS version 20.0 statistical software (IBM Corp., Armonk, NY). A *P* value < 0.05 was considered statistically significant.

## 3. Results

### 3.1. Participants' Characteristics

During the study period, 50 patients met the inclusion criteria with a mean follow-up of 16.8 ± 4.3 months. Patients were of older age (68.4 ± 8.3 years), predominantly male (68.0%), and with a long duration of diabetes (98.1 ± 50.7 months) and severe limb ischemia (ABI: 0.57 ± 0.09). The reasons for nonreconstructable PAD included unpropitious vascular anatomy (62.0%), calcific and fibrocalcific disease in the distal vasculature (28.0%), and patient refusal (10%). All patients at that time were considered candidates for major amputation. There were 22 patients in the conventional group and 28 in the PMMA group. The demographics of the study population are shown in Table 1.

Patients in the PMMA group had a significantly longer duration of DFU (7.9 ± 1.8 months) than those in the conventional group (6.5 ± 2.0 months, *P* = 0.008). Although there were much more severe DFUs (Wagner 4) in the PMMA group, the difference was not statistically significant compared with that in the conventional group (67.9% and 40.9%, respectively, *P* = 0.057). Seven patients (31.8%) used tobacco in the conventional group, which was significantly fewer than the 17 smokers (60.7%) in the PMMA group (*P* = 0.042). Other demographics and clinical characteristics were similar between the two groups.

### 3.2. Microbiological Etiology

All DFUs were infected on initial presentation, and the organism was isolated during debridement. *Staphylococcus aureus* was the most frequently isolated organism, followed by *Pseudomonas aeruginosa*, *Escherichia coli*, and *Enterococcus faecalis*. Methicillin-resistant *Staphylococcus aureus* was present in 5 cases. Cultivation findings were not significantly different between the groups (Table 2).

### 3.3. Outcomes

**Figure 2:**
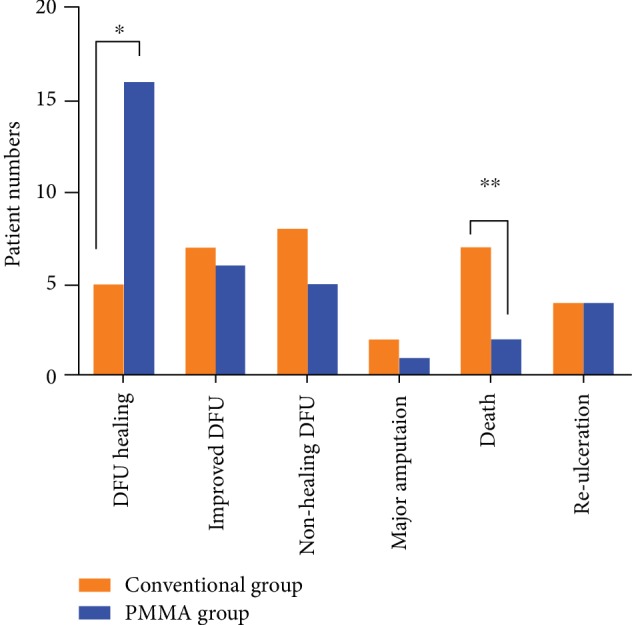
The primary and secondary outcomes between the conventional group and PMMA group (^∗^*P* = 0.014; ^∗∗^*P* = 0.032).

Statistical differences in overall clinical outcomes were observed between the groups (*Z* = −2.495, *P* = 0.013) and are illustrated in Figure 2. When the primary and secondary outcomes were stratified, we found the following data.

In the PMMA group, the leukocyte count and C-reactive protein during the second stage were significantly lower than those in the initial presentation (6.8 ± 1.6 vs. 10.7 ± 4.8, *P* ≤ 0.001, and 11.5 ± 6.9 vs. 71.2 ± 59.8, *P* ≤ 0.001). Although the erythrocyte sedimentation rate showed a decreasing tendency, the difference was nonsignificant (55.5 ± 17.6 vs. 60.3 ± 23.7, *P* = 0.395).

In the PMMA group, 16 patients (57.1%) who had intact IM formation achieved ulceration healing at 13.1 ± 3.7 weeks with a mean number of debridement procedures of 1.3 ± 0.4. Five patients (22.7%, *P* = 0.014) in the conventional group needed a much longer healing duration (26.4 ± 7.8 weeks, *P* = 0.016) and more debridement procedures (3.6 ± 0.5, *P* ≤ 0.001) to achieve healing of DFUs.

During the follow-up period, 6 patients (21.4%) had improved DFUs without clinical signs of infection after a mean number of debridement procedures of 2.2 ± 0.4 in the PMMA group. Of these, the IM was intact in 2 cases and showed regional formation in the remaining 4 patients. Although the number of patients with improved DFUs was similar (*n* = 7, 31.8%, *P* = 0.406), these patients in the conventional group underwent many more interventions (3.7 ± 0.8, *P* = 0.001).

Nonhealing DFUs were observed in 5 patients (17.9%) in the PMMA group and 8 (36.4%, *P* = 0.139) in the conventional group, with mean numbers of debridement procedures of 2.8 ± 0.4 and 3.4 ± 0.5 (*P* = 0.066), respectively. None of these patients in the PMMA group had detectable IM formation.

Major amputation was performed in 3 patients, and all of them were below the knee. Of these, 1 patient (3.6%) in the PMMA group was amputated at 7 months, and 2 patients (9.1%, *P* = 0.576) in the conventional group were amputated at 6 and 12 months, respectively.

At the end of follow-up, patient survival rates were 92.9% in the PMMA group and 68.2% in the conventional group (*P* = 0.032). When stratifying patients according to the cause of death, the only amputee in the PMMA group died of end-stage renal disease at 2 months after major amputation, and the other patient with improved DFU died of stroke at 10 months. In the conventional group, deaths were caused by the following: cardiovascular reason in 2 patients with improved DFUs, life-threatening limb because of refusal to undergo major amputation in 4 patients, and pneumonia at 4 months after major amputation in 1 patient.

DFUs recurred in 4 patients (14.3%) who had complete healing in the PMMA group, and all of them were on the contralateral side. There were also 4 patients (18.2%, *P* = 0.718) with reulceration in the conventional group, in whom 2 new ulcers were located around the previous healing DFUs and 2 others on the contralateral side.

## 4. Discussion

The main findings from this retrospective study supported part of our hypothesis that when compared to routine debridement, IM formation followed by PMMA implantation is effective for wound healing with significantly fewer frequencies of debridement and a shorter duration of healing time in patients with infected DFUs and nonrevascularized PAD. Although the major amputation rate was similar between the groups, the patient survival rate was markedly higher in the PMMA group during a mean follow-up of 16.8 months.

The management of DFU in combination with PAD is of great challenge. The effects of PAD on wound healing include macrovascular and microvascular dysfunction, impaired formation of collateral vessels, and comorbidities mentioned earlier [27]. Data from the EURODIALE Study of 1088 patients with a new DFU in 14 diabetic foot centers demonstrated that PAD conferred poor outcomes within the 1-year follow-up, and the authors suggested that DFU with or without concomitant PAD should be defined as two separate disease states [28]. When revascularization surgery is not feasible, it renders a significant worse prognosis. Marston et al. [29] evaluated the natural history of limbs with PAD and chronic ulceration treated without revascularization and found that major amputation was necessary in 23% of limbs at 12 months. In another study, the 1-year mortality rate was 54%, and only 28% of patients were alive and not amputated at 12 months [30]. However, both studies included some nondiabetic patients. In a recent interdisciplinary study of patients with DFU and nonrevascularized PAD, although only 26% of patients had severe ulcers (Wagner grade ≥ 3), Elgzyri et al. [31] found that at least 17% of patients underwent major amputation, corresponding to one-third of patients who died without DFU healing within a median time of 29 weeks.

Our study enrolled patients who had severe DFUs (Wagner grade 3 or 4) and were not candidates for vascular intervention and reported a notably lower major amputation rate (9.1%) in patients treated with routine debridement than that in the literature. We considered that this discrepancy was attributable to the cultural differences between the East and West regarding the primary goal of limb salvage. It is plausible that patients in Western countries usually regard ambulant ability as the final desired result, whereas elderly Chinese patients have a strong cultural belief to preserve their body and keep it as intact as possible [32]. This belief makes it extremely difficult for most patients in our country to accept major amputation, even when their life is threatened. Accordingly, 4 patients in the conventional group refused to undergo major amputation and died of a life-threatening limb with unhealed DFUs.

Over the past decade, local antibiotic therapy has become an emerging modality in the management of infected DFUs [33, 34]. In theory, the optimal local antibiotic agent should be selected based on the susceptibility patterns of the causative microorganisms at the site of interest. Since urgent surgical intervention is usually required to control infection and the time lapse is present between the specimen culture and pathogen identification, it is difficult to obtain a culture antibiogram preoperatively. Gram-negative bacteria account for more than half of all isolates and the *Staphylococcus* genus is the most frequently observed pathogen in nearly every series in the literature [35, 36], as well as in our study (Table 2). A recent study revealed that vancomycin was one of the most susceptible broad-spectrum antibiotics for the *Staphylococcus* genus and other Gram-positive microorganism isolated from infected DFUs in our country [37]. As a hypoallergenic antibiotic agent, vancomycin also has advanced physicochemical properties of water solubility on diffusion and thermal stability during polymerization, which is available in local antibiotic delivery systems. Therefore, we impregnated vancomycin in the PMMA cement premixed with gentamycin to cover both Gram-positive and Gram-negative bacteria.

Two different types of vehicles, biodegradable and nonbiodegradable, have been used for local antibiotic delivery. As the main representative of a nonbiodegradable substance, the implantation of PMMA to fill anatomical defects secondary to surgical debridement is the standard approach for chronic osteomyelitis and bone defects in orthopedic literature [38, 39]. Although the safety and effectiveness of the local antibiotic delivery system have been recognized in treating DFUs, data on antibiotic-loaded PMMA are sparse and only focus on its antibacterial role [33, 40] or structural supporting capability [41]. Instead of PMMA, calcium sulfate seems to be a preferable carrier in patients with DFUs owing to its biodegradable characteristics and avoidance of further surgery to remove it after infection remission [33, 34]. Therefore, it is not surprising that the additional product from antibiotic-loaded PMMA implantation, the IM, has not been reported in the management of DFUs. On the other hand, compared with the thick, white encapsulation membrane in the typical two-staged approach in bone reconstruction [21, 42], the IM in our study was a thin, semitransparent pseudosynovial membrane (Figure 1) that could be easily confused with surrounding tissues or destroyed during removal of the PMMA spacer. This factor may be another reason why the IM was always neglected in previous studies on DFUs.

Therefore, we conducted this retrospective study to evaluate the effectiveness of the two-staged approach, including vancomycin-loaded PMMA implantation and IM formation, in patients with DFU and PAD. Our results showed that the intact or regional presence of the IM corresponded with complete healing or improved DFUs. Since all patients in our study were not candidates for vascular intervention, we supposed that the IM had a positive impact on local angiogenesis and wound healing. Previous studies demonstrated that neovascularization in the IM significantly increased during the first 2 to 4 weeks at the nonosseous subcutaneous site and then clearly decreased over time [23, 43]. Likewise, the defects left by PMMA removal were usually nonosseous in patients with DFUs, so the second stage in our study was performed at 3 weeks after the initial debridement.

It should be noted that meticulous debridement is crucial for the treatment of DFUs, and IM formation is the sign of definitive eradication of infection [44]. Accordingly, patients in the PMMA group required mean numbers of debridement procedures of 1.3 ± 0.4 and 2.2 ± 0.4 to achieve wound healing and improved DFUs, respectively. In contrast, IM formation was not detected in patients who had nonhealing DFUs even after a mean number of debridement procedures of 2.8 ± 0.4. Moreover, the effect of the IM is topical, and its angiogenesis capability would not be expected to improve limb perfusion when revascularization surgery is not feasible. Thus, the recurrence of DFU had no statistical significance between the PMMA group and conventional group.

The first limitation of our study was the retrospective design. To the best of our knowledge, this is the first study to evaluate the value of IM formation followed by PMMA implantation in the management of DFUs. Although the two-staged approach has been proven to be a good strategy with fairly predictable outcomes in orthopedic surgery, we consider that it is necessary to ensure its safety and validity for treating DFUs before conducting a randomized controlled trial. Second, it could be interpreted by the inclusion criteria in this study that the study population was relatively small, as only patients with severe DFUs (Wagner grade 3 or 4) owing to nonrevascularized PAD were enrolled. During the study period, all patients were aware that the usual option at that time was an unacceptable major amputation for them, and they agreed to undergo this two-staged approach. Third, the follow-up was short. However, these patients have a high short-term mortality rate because of the presence of nonrevascularized PAD; thus, the fundamental goals should be limb salvage without infected DFUs and survival with high quality of life [45].

The strengths of this study are that all perioperative care was performed in the same manner by our multidisciplinary team, and all operative procedures were performed by the same surgeon with the same surgical team, thus minimizing the possibility of therapeutic bias between the two groups.

## 5. Conclusions

The two-staged approach, comprising vancomycin-loaded PMMA implantation and IM formation, is a feasible, effective modality for wound healing in patients with infected DFUs and nonrevascularized PAD. Since this strategy provides promising results in our “no-option” patients, we consider that further study should be required to determine its benefits for patients with or without PAD.

## Figures and Tables

**Table 1:** Baseline demographics and clinical characteristics of the study population.

Variable	Conventional group (*n* = 22)	PMMA group (*n* = 28)	*P* value
Age (mean ± SD, years)	67.8 ± 7.4	68.9 ± 9.1	0.656
Gender			0.558
Male	14 (63.6%)	20 (71.4%)	
Female	8 (36.4%)	8 (28.6%)	
Type 2 DM	20 (90.9%)	24 (85.7%)	0.683
DM duration (mean ± SD, months)	96.3 ± 55.9	99.6 ± 47.3	0.822
DFU duration (mean ± SD, months)	6.5 ± 2.0	7.9 ± 1.8	**0.008**
Wagner classification			0.057
Grade 3	13 (59.1%)	9 (32.1%)	
Grade 4	9 (40.9%)	19 (67.9%)	
ABI (mean ± SD)	0.58 ± 0.10	0.57 ± 0.08	0.620
Nonreconstructable reasons			0.629
Unpropitious vascular anatomy	14 (63.6%)	17 (60.7%)	
Calcific and fibrocalcific disease	5 (22.7%)	9 (32.1%)	
Patient refusal	3 (13.6%)	2 (7.1%)	
BMI (mean ± SD, kg/m^2^)	26.3 ± 4.5	25.2 ± 4.1	0.391
Smoking	7 (31.8%)	17 (60.7%)	**0.042**
Alcohol abuse	7 (31.8%)	11 (39.3%)	0.585
Follow-up (mean ± SD, months)	16.4 ± 4.6	17.1 ± 4.1	0.575
Laboratory findings (mean ± SD)			
Glycemia (% (mmol/L))	11.4 ± 6.1	11.0 ± 5.4	0.781
HbA_1C_ (% (mmol/mol))	7.5 ± 1.4	7.8 ± 1.6	0.449
WBC (×10^9^/L)	9.6 ± 5.9	10.7 ± 4.8	0.467
CRP (*μ*mol/L)	63.6 ± 51.7	71.2 ± 59.8	0.639
ESR (mm/hr)	55.8 ± 24.1	60.3 ± 23.7	0.518
Albumin (g/L)	29.9 ± 10.6	28.7 ± 10.1	0.687
Comorbidities			
Hypertension	13 (59.1%)	21 (75.0%)	0.231
Ischemic heart disease	9 (40.9%)	14 (50.0%)	0.522
Peripheral neuropathy	20 (90.9%)	23 (82.1%)	0.444
Nephropathy	13 (59.1%)	15 (53.6%)	0.696
Retinopathy	10 (45.5%)	16 (57.1%)	0.412

PMMA: polymethylmethacrylate; DM: diabetes mellitus; DFU: diabetic foot ulcer; ABI: ankle-brachial index; BMI: body mass index; WBC: white blood cell; CRP: C-reactive protein; ESR: erythrocyte sedimentation rate. Values are *n* (%) unless otherwise noted. Boldface indicated statistically significant difference.

**Table 2:** Microbiological findings.

Microbiological findings	Overall	Conventional group	PMMA group
*Staphylococcus aureus*	24	11	13
*Pseudomonas aeruginosa*	15	6	9
*Escherichia coli*	8	5	3
*Enterococcus faecalis*	8	4	4
*Enterobacter cloacae*	8	3	5
MRSA	5	2	3
*Candida tropicalis*	3	2	1

PMMA: polymethylmethacrylate; MRSA: methicillin-resistant *Staphylococcus aureus*. Values are numbers of isolated pathogens.

## Data Availability

The data used to support the findings of this study are available from the corresponding authors upon request.
